# Survival outcomes in esophageal cancer patients with a prior cancer

**DOI:** 10.1097/MD.0000000000024798

**Published:** 2021-02-19

**Authors:** Deqiang Pan, Wenbo Xu, Xingcai Gao, Feng Yiyang, Shuai Wei, Guang Zhu

**Affiliations:** Department of Surgery, The Fifth Affiliated Hospital of Zhengzhou University, Zhengzhou, China.

**Keywords:** esophageal cancer, second primary malignancy, surveillance, epidemiology, and end result, survival

## Abstract

To achieve a deeper understanding of patients who developed esophageal cancer (EC) as a second primary malignancy, which may help guide in clinical practice for these patients in the future.

In the primary cohort, EC patients with a prior malignancy were identified from the surveillance, epidemiology, and end result 18 database. The 5 most common types of prior cancers were picked out based on the frequency of occurrence. In addition, Kaplan–Meier and log-rank tests were performed to investigate the survival impacts of prior cancers on EC patients. Besides, a competing-risk model was constructed to explore the relationship between EC-treatment and EC-specific mortality. In the secondary cohort, patients with stage I–III (N0M0) EC from 2004 to 2014 were enrolled. After propensity score matching, univariate and multivariate Cox analyses were developed to determine the prognostic factors for EC patients.

A total of 1199 EC patients with a prior cancer were identified in the primary cohort. The 5 most common sites of prior cancers were prostate, female breast, bladder, lung and bronchus, and larynx. Kaplan–Meier analyses revealed that EC patients with prior prostate cancer and bladder cancer had the best overall survival (OS), while those with prior cancers of larynx and lung and bronchus had the worst OS. Fine and Gray competing risks analysis indicated that the administration of surgery was closely associated with better EC-specific survival (*P* < .001). In the secondary cohort, multivariate Cox analyses found that age at diagnosis, race, tumor grade, tumor extent, nodal status and metastasis stage, histology, and the administration of surgery were prognostic factors for OS and cancer-specific survival in EC patients. Besides, the existence of a prior cancer was an independent prognostic factor for cancer-specific survival.

EC remains to be the most important cause of death in EC patients with a prior cancer. EC related treatment should be actively adopted in patients with a prior cancer, as they were more likely to die from EC than the prior cancer. EC patients with a prior cancer had comparable OS than those without.

## 1 Introduction

Esophageal cancer (EC) is one the most common malignancies, the incidence rate (IR) ranked ninth of all malignant tumors worldwide in 2018.^[1]^ In 2020, the estimated new cases and deaths were 18,440 and 16,170 in the United States (US).^[2]^ Surgery and radiotherapy have been the standard treatment types of EC for many years. Nowadays, rapid development of immunotherapy and targeted therapy (such as trastuzumab) of EC has brought a tremendous promise in the treatment of EC.^[3,4]^ Moreover, the 5-year survival rate of EC patients has increased from 10% to 25% due to the advancement of cancer detection and treatment.^[5,6]^ Hence, more and more cancer survivors developed a second primary malignancy (SPM) because of the increasing IRs and improvement of survival outcomes.^[7,8]^

SPM is defined as a cancer which develops in a new tissue or organ after the initial diagnosis of the prior malignancy with a 6-month latency. Previous studies mainly focused on the risk of developing an SPM after a known malignancy. Liao et al^[9]^ discussed the main prognostic factors for oral cavity cancer patients with simultaneous SPM, and then developed a risk-stratification. Vassilev et al^[10]^ provided a historical risk estimation of developing an SPM in patients with metastatic castration-resistant prostate cancer. However, as far as we know, survival outcomes of patients with 1 known tumor as an SPM have not been well studied. Only a few published studies have discussed the risk of developing an SPM in primary cancer survivors.^[11,12]^ Saad et al^[13]^ investigated the impact of the prior cancer on survival outcomes of stage IV EC patients, they found that prior cancers did not adversely impact survival of EC patients with stage IV diseases. Besides, Chen et al^[14]^ explored clinicopathological characteristics and prognosis of patients with EC as an SPM, they demonstrated that lower M stage, the administration of surgery, and chemotherapy were tightly related to better overall survival (OS) for patients with EC as an SPM.

In this study, patients diagnosed with EC as an SPM were extracted from the surveillance, epidemiology, and end result (SEER) database retrospectively. We aimed to achieve a deeper understanding of the outcomes of patients who developed EC as an SPM, which may help guide in clinical practice for these patients in the future.

## 2 Materials and methods

### 2.1 Database

Data were extracted from the SEER database retrospectively. It is a population-based registry sponsored by the US National Cancer Institute. The SEER database collects relevant information of cancer IR, baseline characteristics, treatment types and long-term follow-up, and covers approximately 34.6% of the US population till now (https://seer.cancer.gov/about/overview.html). We signed the Research Data Agreement before this study and got access to the database with the username of 11015-Nov2019. In addition, use of SEER registry was exempt by Institutional Review Board approval.

### 2.2 Primary cohort

In this section, we extracted EC patients with a prior malignancy from the SEER 18 program using the “multiple primary-standard incidence ratio” function by the SEER∗Stat software (version 8.3.6; US National Cancer Institute, Bethesda, Maryland, USA). EC was diagnosed as the SPM with positive pathology. Furthermore, the exclusion criteria were as follows:

(1) patients with more than 2 malignancies in total,
(2) data were from autopsy or death certificate only,
(3) year of diagnosis was not from 2004 to 2014,
(4) patients with missing or unknown data,
(5) interval between diagnosis of EC and the prior cancer was less than 6 months.

A 6-month latency was utilized to distinguish SPMs from simultaneous cancers. In our study, the International Classification of Diseases for Oncology third edition primary site codes for EC contained C15.0 (cervical esophagus), C15.1 (thoracic esophagus), C15.2 (abdominal esophagus), C15.3 (upper third of esophagus), C15.4 (middle third of esophagus), C15.5 (lower third of esophagus), C15.8 (overlapping lesion of esophagus), and C15.9 (esophagus, not otherwise specified). To be specific, codes C15.0 and C15.3 were used to identify upper esophageal tumors, while C15.4 was for middle esophageal tumors and C15.2 and C15.5 were for lower esophageal tumors. Moreover, histologic recode broad groupings were applied for the classification of histological subtypes (codes 8140-8389 were for adenomas and adenocarcinomas (AC), codes 8050-8089 were for squamous cell carcinomas (SCC) and all other remaining codes as other histology.

Then, demographic characteristics and clinical data for each patient were collected, including age at diagnosis (both prior cancer and EC), sex, race, histological type, primary sites of EC, American Joint Committee on Cancer 6th tumor extent, nodal status and metastasis (TNM) stage, diagnosis intervals, the administration of surgery, radiotherapy and chemotherapy, vital status, cause of death (COD) and follow-up. Age at diagnosis was categorized into <65 and ≥65 years old. Furthermore, CODs were classified into 3 groups: died from EC, died from the prior cancer, and died from other causes.

First of all, we picked out the 5 most common types of prior cancers based on the frequency of occurrence. Then, Kaplan–Meier and log-rank tests were performed to investigate the survival impacts of prior cancers on EC patients. Afterward, the percentage of EC-related and prior cancer-related deaths in patients with different prior malignancies were calculated, and the ratios of EC deaths to prior cancer deaths were obtained, further stratified by EC TNM stage and histological type. Finally, to explore the relationship between the administration of surgery and EC-specific mortality (ECSM), we constructed a competing model after taking died from other causes/prior cancers as a competing event.

### 2.3 Secondary cohort

In the secondary cohort, we identify patients with stage I-III (N0M0) EC from 2004 to 2014 in the SEER 18 database using the “case listing session” function. Based on the existence of a prior malignancy, all patients were then divided into “primary esophageal cancer (PEC)” and “subsequent esophageal cancer (SEC).” Propensity score matching (PSM) method was used to balance the basic characteristics of PEC and SEC patients with a ratio of 1:1. Survival discrepancies between PEC and SEC patients were compared before and after PSM. Lastly, univariate and multivariate Cox analyses were developed to discuss the prognostic factors which were significantly related to OS and cancer-specific survival (CSS) in patients with EC.

### 2.4 Statistical analysis

Student *t* test and Mann–Whitney *U* test were used for the comparisons of continuous variables. Chi-square analysis was utilized to make comparisons between categorical variables. The whole analysis was based on SPSS 23.0 (SPSS Inc, Chicago, IL) and R software (Version 3.4.1). A 2-sided *P* < .05 was considered significant.

## 3 Result

### 3.1 Baseline characteristics of the primary cohort

A total of 1199 EC patients with a prior cancer were eventually enrolled in the primary cohort. As shown in Table 1, the median (interquartile range [IQR]) ages at EC and the prior cancer diagnosis were 73.00 (66.00–80.00) and 64.00 (57.00–71.00) years old, respectively. Most patients were White (85.99%) and male (78.73%). The most common site of EC was lower esophagus (61.38%). 54.38% of the EC patients were with AC. The median (IQR) diagnosis interval between the prior cancer and EC was 91.00 (43.99–151.00) months. Moreover, the median (IQR) follow-up since EC diagnosis was 12.00 (4.00–30.00) months.

**Table 1:** Demographic and clinical factors of EC patients with a prior cancer (n = 1199).

Variables	Value
At prior cancer diagnosis	
Age, yr	
Mean (SD)	63.23 (11.65)
Median (IQR)	64.00 (57.00–71.00)
Sex, n (%)	
Male	944 (78.73)
Female	255 (21.27)
Race, n (%)	
White	1031 (85.99)
Black	111 (9.26)
Other	57 (4.75)
At EC diagnosis	
Age, yr	
Mean (SD)	72.46 (9.99)
Median (IQR)	73.00 (66.00–80.00)
Primary site, n (%)	
Upper	129 (10.76)
Middle	193 (16.10)
Lower	736 (61.38)
Other	141 (11.76)
Histology, n (%)	
AC	652 (54.38)
SCC	453 (37.78)
Other	92 (7.67)
TNM stage, n (%)	
I-II	634 (52.88)
III-IV	565 (47.12)
Interval between diagnoses, mo	
Mean (SD)	110.77 (87.27)
Median (IQR)	91.00 (43.00–151.00)
Time from EC diagnosis to death or end of study, mo	
Mean (SD)	22.76 (27.67)
Median (IQR)	12.00 (4.00–30.00)

### 3.2 Survival outcomes in the primary cohort

**Figure 1:**
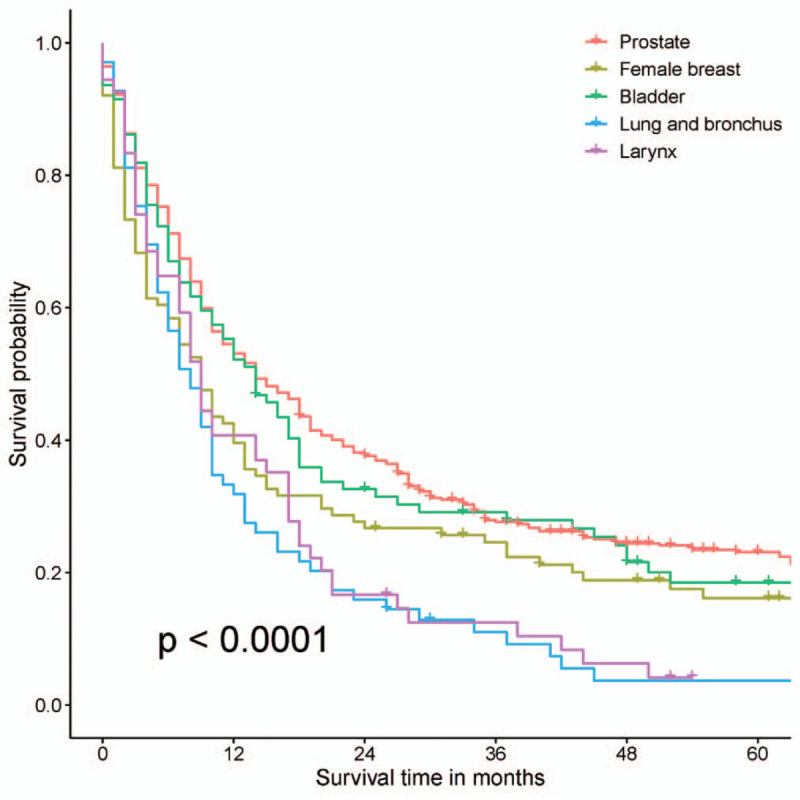
Overall survival of esophageal cancer patients with a prior cancer.

The 5 most common sites of prior cancers were prostate (35.36%), female breast (8.42%), bladder (7.84%), lung and bronchus (5.75%), and larynx (4.50%) (Table 2). OS was significantly different in EC patients with different prior malignancies (*P* < .0001, Fig. 1). EC patients with prior prostate cancer and bladder cancer had the best survival outcomes (3-year OS rates were 27.7% and 29.2%, respectively), while those with prior cancer of larynx and lung and bronchus had the worst OS (3-year OS rates were 12.5% and 11.0%, respectively).

**Table 2:** Classification of the prior malignancy.

Sites	N (%)	Death, n (%)
Prostate	424 (35.36)	344 (81.13)
Female breast	101 (8.42)	86 (85.15)
Bladder	94 (7.84)	76 (80.85)
Lung and bronchus	69 (5.75)	65 (94.20)
Larynx	54 (4.50)	51 (94.44)
Overall	1199 (100)	1009 (84.15)

**Figure 2:**
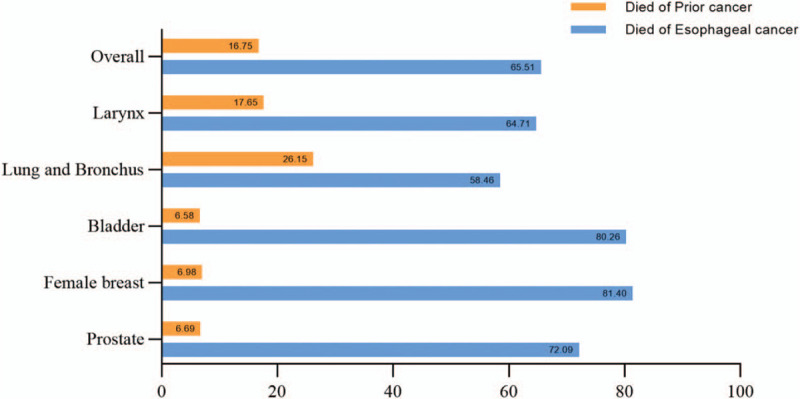
Distribution of causes of death in the top 5 most common sites of developing SPMs in EC patients. EC = esophageal cancer, SPM = second primary malignancy.

**Figure 3:**
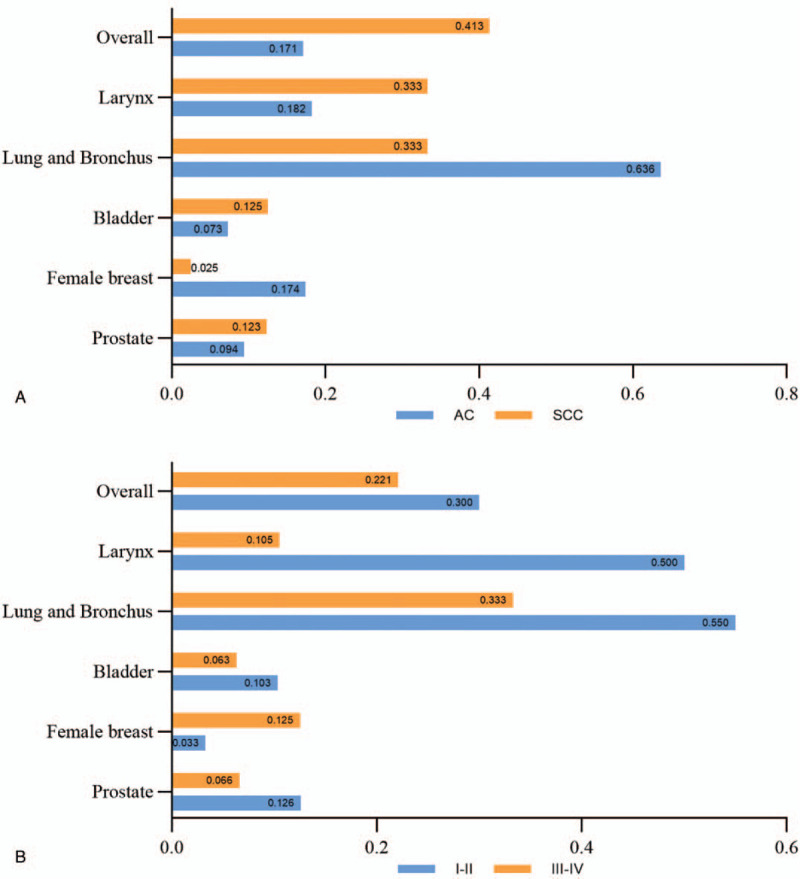
The ratios of prior cancer-related deaths to EC-related deaths. EC = esophageal cancer.

In the analysis of COD, 65.51% of EC patients died from EC and 16.75% of patients died from the prior cancer (Fig. 2). EC patients with prior cancers of lung and bronchus had the highest prior cancer-related death rate (26.15%) and the lowest EC-related death rate (58.46%). Furthermore, the ratios of prior cancer-related deaths to EC-related deaths were calculated. As shown in Figure 3, the ratios were less than 1 regardless of the histological type (Fig. 3A) or TNM stage (Fig. 3B) of EC. Hence, conclusion could be drawn that EC patients were more likely to die of EC regardless of the cancer types of prior cancers and EC.

**Figure 4:**
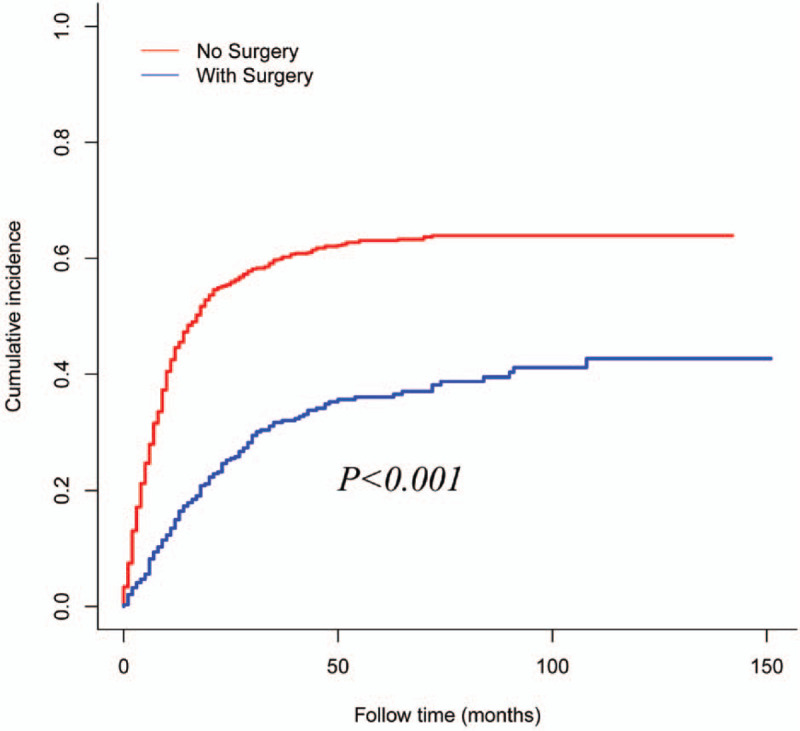
Estimates of overall cumulative incidence of developing a second malignancy, taking surgery as a competing event.

Compared with patients who died from the prior cancer, those who died from EC had older ages at cancer diagnosis (both EC and the prior cancer) (all *P* < .05, Table 3). In addition, the proportions of AC and N1 diseases (all for EC) were significantly higher in patients who died from EC. The median interval between diagnosis of 2 cancers was significantly longer in patients who died from EC than that in patients who died from the prior cancer (92.00 vs 66.00 months, *P* < .001). Notably, the percentage of radiotherapy in patients who died from EC was significantly higher than those who died from the prior cancer (62.93% vs 53.85%, *P* = .031). To explore the prognostic role of cancer treatments, Fine and Gray competing risks analyses were developed. As shown in Figure 4, the administration of surgery was tightly related to better EC-specific survival (*P* < .001).

**Table 3:** Clinical and demographic factors associated with EC death versus prior cancer death.

Characteristics	Died from prior cancer	Died from EC	*P-*value
Number of patients	169	661	
Age at EC diagnosis, median (IQR), yr	70.00 (62.00–76.00)	75.00 (67.00–81.00)	***<.001***
Age at prior cancer diagnosis, median (IQR), yr	62.00 (54.00–70.00)	65.00 (57.50–72.00)	***.021***
EC, histology, n (%)			***<.001***
AC	59 (34.91)	345 (52.19)	
SCC	104 (61.54)	252 (38.12)	
Other	6 (3.55)	64 (9.68)	
EC, surgery treated, n (%)			*.572*
No	133 (78.70)	533 (80.64)	
Yes	36 (21.30)	128 (19.36)	
EC, radiotherapy treated, n (%)			***.031***
No/unknown	78 (46.15)	245 (37.07)	
Yes	91 (53.85)	416 (62.93)	
EC, chemotherapy treated, n (%)			*.074*
No/unknown	74 (43.79)	240 (36.31)	
Yes	95 (56.21)	421 (63.69)	
EC, TNM stage, n (%)			*.063*
I-II	91 (53.85)	303 (45.84)	
III-IV	78 (46.15)	358 (54.16)	
EC, Tx/N1/Mx, n (%)	71 (42.01)	351 (53.10)	***.010***
EC, Tx/Nx/M1, n (%)	42 (24.85)	194 (29.35)	*.247*
EC, grade I-II, n (%)	127 (75.15)	537 (81.24)	*.077*
Interval between diagnoses, median (IQR), mo	66.00 (32.50–112.00)	92.00 (48.00–163.50)	***<.001***

### 3.3 Survival of patients with EC as the prior cancer or subsequent primary cancer in the second cohort

From 2004 to 2014, a total of 7230 patients with stage I–III EC were enrolled in the secondary cohort, including 5281 (73.04%) patients had EC as the only malignancy (PEC) and 1949 (26.96%) patients with EC following a prior cancer (defined as SEC) (Table 4). SEC patients had significantly older age than PEC patients (≥65 years old: 77.68% vs 58.97%, *P* < .001). Furthermore, the proportions of male patients, lower esophageal tumors, histology of AC, higher stage (II–III) diseases, and the administration of surgery/radiotherapy/chemotherapy were significantly higher in PEC patients when compared with these in SEC patients (all *P* < .05). Therefore, a 1:1 PSM was applied to minimize the difference between SEC and PEC patients in baseline characteristics and treatment types. Eventually, a total of 1949 pairs of EC patients were included.

**Table 4:** Baseline characteristics of patients with PEC or SEC from the SEER database 2004-2014.

	Data before PSM			Data after PSM		
Variables	PEC	SEC	*P-*value	PEC	SEC	*P-*value
N	5281	1949		1949	1949	
Age (yr)			***<.001***			*.393*
<65	2167 (41.03)	435 (22.32)		413 (21.19)	435 (22.32)	
≥65	3114 (58.97)	1514 (77.68)		1536 (78.81)	1514 (77.68)	
Race			*.112*			*.299*
White	4442 (84.11)	1660 (85.17)		1678 (86.10)	1660 (85.17)	
Black	583 (11.04)	217 (11.13)		190 (9.75)	217 (11.13)	
Other	256 (4.85)	72 (3.69)		81 (4.16)	72 (3.69)	
Sex			***<.001***			*1.000*
Male	4086 (77.37)	1388 (71.22)		1388 (71.22)	1388 (71.22)	
Female	1195 (22.63)	561 (28.78)		561 (28.78)	561 (28.78)	
Location			***<.001***			*.146*
Upper	461 (8.73)	322 (16.52)		278 (14.26)	322 (16.52)	
Middle	1055 (19.98)	444 (22.78)		461 (23.65)	444 (22.78)	
Lower	3765 (71.29)	1183 (60.70)		1210 (62.08)	1183 (60.70)	
Grade^∗^			*.464*			*.702*
Grade I	494 (9.35)	170 (8.72)		155 (7.95)	170 (8.72)	
Grade II	2485 (47.06)	926 (47.51)		928 (47.61)	926 (47.51)	
Grade III	2217 (41.98)	830 (42.59)		837 (42.95)	830 (42.59)	
Grade IV	85 (1.61)	23 (1.18)		29 (1.49)	23 (1.18)	
Histology			***<.001***			*.061*
AC	3156 (59.76)	967 (49.62)		1034 (53.05)	967 (49.62)	
SCC	1772 (33.55)	868 (44.54)		795 (40.79)	868 (44.54)	
Other	353 (6.68)	114 (5.85)		120 (6.16)	114 (5.85)	
TNM stage			***<.001***			*.798*
I	2616 (49.54)	1102 (56.54)		1121 (57.52)	1102 (56.54)	
II	2176 (41.20)	682 (34.99)		671 (34.43)	682 (34.99)	
III	489 (9.26)	165 (8.47)		157 (8.06)	165 (8.47)	
Surgery			***<.001***			*.383*
No	2858 (54.12)	1249 (64.08)		1275 (65.42)	1249 (64.08)	
Yes	2423 (45.88)	700 (35.92)		674 (34.58)	700 (35.92)	
Radiation			***.006***			*.700*
No/unknown	2326 (44.04)	929 (47.67)		917 (47.05)	929 (47.67)	
Yes	2955 (55.96)	1020 (52.33)		1032 (52.95)	1020 (52.33)	
Chemotherapy			***<.001***			*.949*
No/unknown	2487 (47.09)	1036 (53.16)		1034 (53.05)	1036 (53.16)	
Yes	2794 (52.91)	913 (46.84)		915 (46.95)	913 (46.84)	

**Figure 5:**
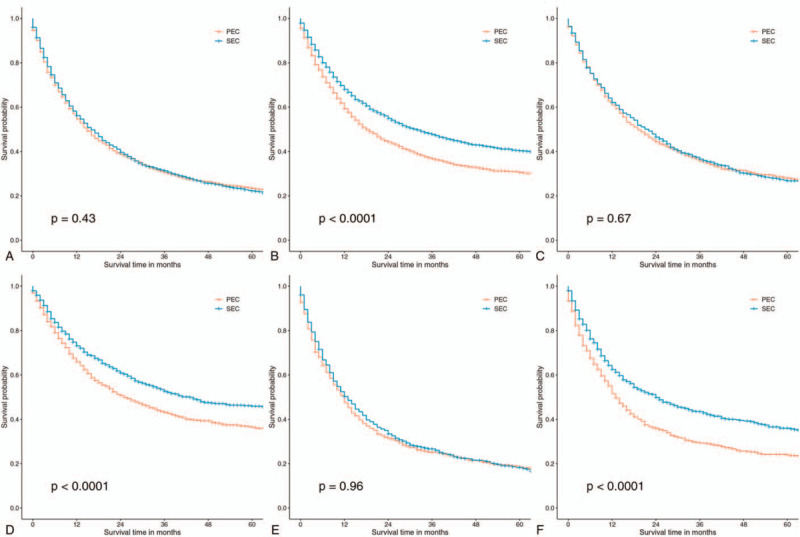
The comparisons of survival outcomes between SEC and PEC patients (after matching). OS in the whole population (A); CSS in the whole population (B); OS in patients with esophageal adenocarcinoma (C); CSS in patients with esophageal adenocarcinoma (D); OS in patients with esophageal squamous cell carcinomas (E); CSS in patients with esophageal squamous cell carcinomas (F). PEC = primary esophageal cancer, SEC = subsequent esophageal cancer.

Supplemental Digital Content (Figure S1) shows the comparisons of survival outcomes between SEC and PEC patients. After matching, there was no significant difference in OS between patients in 2 groups (Fig. 5A, *P* > .05). However, SEC patients had better CSS than PES patients (Fig. 5B, *P* < .05). Furthermore, subgroup analyses based on different histological types (AC and SCC) revealed the same results (Fig. 5C–F).

Multivariate Cox analysis indicated that age at diagnosis, race, tumor grade, TNM stage, histology, and the administration of surgery were prognostic factors for OS and CSS in EC patients (Tables 5 and 6). Besides, the existence of a prior cancer (PEC vs SEC) was an independent risk factor for CSS (*P* < .001).

**Table 5:** Uni- and multivariate Cox regression model analysis of OS.

	Univariate			Multivariate		
Variables	HR	95% CI	*P-*value	HR	95% CI	*P-*value
Age, yr			***<.001***			***<.001***
<65	Reference			Reference		
≥65	1.462	1.337–1.599	*<.001*	1.409	1.286–1.544	*<.001*
Race			***<.001***			***.001***
White	Reference			Reference		
Black	1.450	1.299–1.619	*<.001*	1.205	1.072–1.356	*.002*
Other	0.913	0.757–1.103	*.346*	0.841	0.695–1.071	*.074*
Sex			*.251*			
Male	Reference					
Female	1.047	0.968–1.131	*.251*			
Grade^∗^			***<.001***			***<.001***
Grade I-II	Reference			Reference		
Grade III-IV	1.291	1.203–1.386	*<.001*	1.171	1.088–1.260	*<.001*
TNM stage			***<.001***			***<.001***
I	Reference			Reference		
II	1.047	0.970–1.130	*.236*	0.965	0.893–1.042	*.360*
III	1.914	1.692–2.167	*<.001*	1.532	1.352–1.736	*<.001*
Histology			***<.001***			***.048***
AC	Reference			Reference		
SCC	1.375	1.278–1.479	*<.001*	1.078	0.975–1.192	*.144*
Other	1.413	1.219–1.638	*<.001*	1.191	1.024–1.385	*.024*
Location			***<.001***			*.647*
Upper	Reference			Reference		
Middle	0.957	0.856–1.071	*.446*	1.026	0.916–1.150	*.658*
Lower	0.768	0.696–0.847	*<.001*	1.059	0.937–1.197	*.357*
Diagnosis			*.441*			
PEC	Reference					
SEC	1.028	0.958–1.103	*.441*			
Surgery			***<.001***			***<.001***
No	Reference			Reference		
Yes	0.336	0.310–0.365	*<.001*	0.363	0.333–0.395	*<.001*

**Table 6:** Uni- and multivariate Cox regression model analysis of CSS.

	Univariate			Multivariate		
Variables	HR	95% CI	*P-*value	HR	95% CI	*P-*value
Age, yr			***<.001***			***<.001***
<65	Reference			Reference		
≥65	1.434	1.290–1.595	*<.001*	1.360	1.220–1.515	*<.001*
Race			***<.001***			***.026***
White	Reference			Reference		
Black	1.464	1.287–1.665	*<.001*	1.187	1.034–1.362	*.015*
Other	1.023	0.828–1.264	*.830*	0.907	0.732–1.125	*.375*
Sex			*.121*			
Male	Reference					
Female	1.075	0.981–1.178	*.121*			
Grade^∗^			***<.001***			***<.001***
Grade I-II	Reference			Reference		
Grade III-IV	1.357	1.248–1.475	*<.001*	1.208	1.108–1.317	*<.001*
TNM stage			***<.001***			***<.001***
I	Reference			Reference		
II	1.104	1.009–1.207	*.031*	1.001	0.915–1.097	*.975*
III	2.132	1.852–2.455	*<.001*	1.678	1.455–1.935	*<.001*
Histology			***<.001***			***.041***
AC	Reference			Reference		
SCC	1.416	1.299–1.544	*<.001*	1.117	0.991–1.258	*.069*
Other	1.479	1.245–1.757	*<.001*	1.210	1.014–1.443	*.034*
Location			***<.001***			*.242*
Upper	Reference			Reference		
Middle	1.010	0.885–1.153	*.881*	1.088	0.952–1.245	*.215*
Lower	0.783	0.697–0.880	*<.001*	1.130	0.978–1.306	*.097*
Diagnosis			***<.001***			***<.001***
PEC	Reference			Reference		
SEC	0.762	0.700–0.828	*<.001*	0.740	0.680–0.804	*<.001*
Surgery			***<.001***			***<.001***
No	Reference			Reference		
Yes	0.282	0.254–0.312	*<.001*	0.305	0.274–0.340	*<.001*

## 4 Discussion

In recent years, the number of cancer survivors is rapidly increasing due to the improvement of cancer screening and treatment. Hence, the risk of developing an SPM in cancer survivors has also been increasing.^[7]^ It was reported that there was a 2% annual increase for the cancer survivor population in the US, and about 18% of cancer survivors developed an SPM during the rest of their lifetime according to the SEER registry.^[15]^ Furthermore, the history of a prior cancer played a critical role in making clinical decision, especially for those who participated in clinical trials. In many clinical trials, history of a prior cancer was a strict exclusion criterion for potential candidates, which may be due to the survival impacts of the prior cancers.^[16]^ Although there was no powerful evidence supporting the hypothesis that exclusion of these patients could balance the outcomes and validity of clinical trials,^[13]^ many published trials excluded patients with a prior cancer routinely.^[17–19]^ A previous study revealed that there were approximately 20% of lung cancer patients were excluded because of this restrictive exclusion rule.^[18]^ This study was to investigate the survival outcomes of EC patients with a prior cancer and to identify prognostic factors for EC patients.

In this study, the most common prior malignancy in EC patients was prostate cancer, followed by female breast cancer, bladder cancer, and lung cancer. Interestingly, these cancers are also the most common cancers as single malignancy in general. Hence, we guessed that there was no enrichment for a cancer type that may increase the risk of developing EC as an SPM. Similarly, Zhu et al^[20]^ reported that the most common types of prior cancers in larynx cancer patients were from prostate, lung and bronchus, urinary bladder, and breast. Laccetti et al^[21]^ found that prostate, gastrointestinal, breast, and other genitourinary were the most common types of prior cancer in locally advanced lung cancer.

Comparisons in survival outcomes of EC patients with different prior cancers showed significant statistical difference. EC patients with prior cancers of prostate cancer and bladder cancer had significant better OS than those with prior cancers of lung and bronchus. The survival discrepancy may be due to the level of threat to life of prior cancers. Moreover, EC patients were more likely to die of EC regardless of the cancer types of prior cancers and EC. Lastly, multivariate Cox analyses found that age, race, tumor grade, TNM stage, histology, and the administration of surgery were independent prognostic factors for OS and CSS in EC patients, and the existence of a prior cancer was an independent risk factor for CSS.

Most patients died from EC rather than the prior cancer (65.51% vs 16.75%) with a median follow-up of 12.00 months. Furthermore, subgroup analyses based on TNM stage and histology (AC and SCC) revealed the same results. Moreover, Kaplan–Meier analysis showed that PEC patients had similar OS compared with SEC patients. Saad et al^[13]^ found that stage IV EC patients with a prior cancer had comparable OS with those had EC as their only malignancy. In that study, Saad et al only focused on the survival impact of prior cancers on the advanced EC patents, rather than all EC patients. Similarly, Chen et al^[14]^ investigated the clinicopathological characteristics and survival outcomes of EC patients with a prior cancer, they found that the most common prior malignancy in EC patients was from genital system (about 43.5%). Moreover, EC patients with a prior cancer had comparable OS when compared with only primary EC patients. However, previous studies did not investigate the EC-specific survival. In our study, SEC patients had significant better CSS than PEC patients after matching. Better CSS could be attributed to the fact that cancer survivors receiving a stricter screening and care or being more cautious on healthy problems. Furthermore, Wang et al^[22]^ reported that nasopharyngeal carcinoma patients with a prior cancer had better CSS than those without a prior cancer. However, study conducted by Ji et al^[23]^ and Al-Husseini et al^[24]^ reached the opposite conclusions that breast cancer or glioblastoma patients with a prior malignancy had worse CSS than those had breast cancer or glioblastoma as their only malignancy.

In our study, the proportion of surgery was comparable in patients who died from EC with that in patients who died from the prior cancer. Interestingly, Fine and Gray competing analysis showed that the administration of surgery was closely related to a reduction of ECSM. Our findings strongly indicated that surgery was still an optional alternative for EC patients with a prior cancer. First, most EC patients with a prior cancer died from EC rather than the prior cancer, regardless of the clinical characteristics of the prior cancer and EC. Second, prolonged CSS was detected in SEC patients when compared with PEC patients. Dinh et al^[12]^ found that treatment for patients with high stage and high-grade prostate cancer was related to a decreased risk of prostate cancer-specific mortality.

Cox regression analyses revealed that age at diagnosis, race, tumor grade, TNM stage, histology, and the administration of surgery were prognostic factors for OS and CSS in EC patients. Many previous studies have explored the prognostic factors for OS and CSS in cancer survivors. Traditionally, age at diagnosis, tumor grade, TNM stage, and the administration of surgery were widely recognized risk factors for survival in many cancer types. In our study, the existence of a prior cancer (PEC vs SEC) was identified to be an independent prognostic factor for CSS, but not for OS. Some studies demonstrated that a prior cancer could seriously affect the survival of cancer survivors, and those with prior malignancies should be excluded from clinical trials. However, our data supported that careful selection of candidates for clinical trials should be performed in EC patients with a prior cancer, rather than excluding all patients.

However, there were some limitations that should not be ignored. First, numerous data were lacking or missing in the SEER registry. Second, the nature of retrospective research led to the inevitable selection bias. Moreover, treatment strategies of prior cancers may have something to do with the occurrence and survival of SPM.^[25,26]^ Therefore, further prospective and well-designed studies are needed to validate our findings.

## 5 Conclusions

In EC patients with a prior cancer, EC is the most important COD regardless of the clinical characteristics of the prior cancer and EC. Surgery for these patients decreased the risk of ECSM. These finding suggested that EC related treatment should be actively adopted in patients with prior cancers, as they were more likely to die from EC than the prior cancer. Lastly, age at diagnosis, race, tumor grade, TNM stage, histology, and the administration of surgery were found to be prognostic factors for OS and CSS in EC patients.

## Author contributions

**Conceptualization:** Deqiang Pan, Guang Zhu.

**Data curation:** Deqiang Pan, Wenbo Xu, Xingcai Gao, Feng Yiyang, Shuai Wei.

**Formal analysis:** Deqiang Pan, Wenbo Xu, Guang Zhu.

**Methodology:** Wenbo Xu.

**Writing – original draft:** Deqiang Pan.

**Writing – review & editing:** Deqiang Pan, Wenbo Xu, Xingcai Gao, Feng Yiyang, Shuai Wei.

## Supplementary Material

Supplemental Digital Content
