# Supplementary material for: Survival outcomes in esophageal cancer patients with a prior cancer
Source: Medicine (Baltimore). 2021 Feb 19;100(7):e24798. doi: 10.1097/MD.0000000000024798 (PMC7899859; doi:10.1097/MD.0000000000024798)
Supplement: Supplemental Digital Content [file medi-100-e24798-s001.doc]

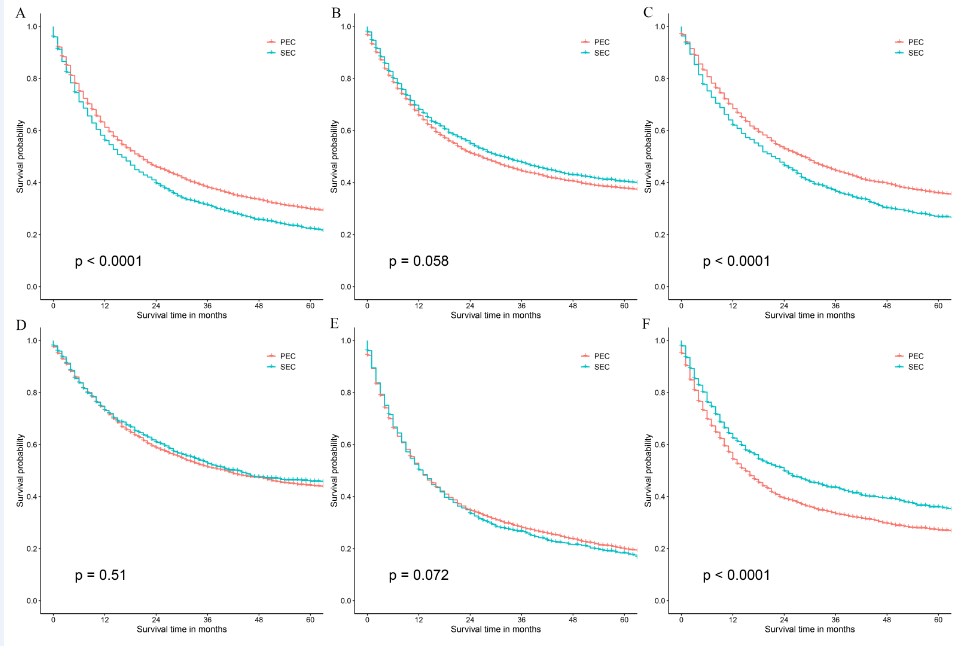


Figure S1: The comparisons of survival outcomes between SEC and PEC patients (before matching). OS in the whole population (A); CSS in the whole population (B); OS in patients with esophageal adenocarcinoma (C); CSS in patients with esophageal adenocarcinoma (D); OS in patients with esophageal squamous cell carcinomas (E); CSS in patients with esophageal squamous cell carcinomas (F). SEC: subsequent esophageal cancer, PEC: primary esophageal cancer.
